# Early EEG monitoring predicts clinical outcome in patients with moderate to severe traumatic brain injury

**DOI:** 10.1016/j.nicl.2023.103350

**Published:** 2023-02-14

**Authors:** Prejaas K.B. Tewarie, Tim M.J. Beernink, Carin J. Eertman-Meyer, Alexander D. Cornet, Albertus Beishuizen, Michel J.A.M. van Putten, Marleen C. Tjepkema-Cloostermans

**Affiliations:** a Clinical Neurophysiology Group, University of Twente, Enschede, the Netherlands; b Department of Neurology and Clinical Neurophysiology, Medisch Spectrum Twente, Enschede, the Netherlands; c Department of Neurology, Amsterdam UMC/VUmc, Amsterdam, the Netherlands; d Intensive Care Center, Medisch Spectrum Twente, Enschede, the Netherlands

## Abstract

There is a need for reliable predictors in patients with moderate to severe traumatic brain injury to assist clinical decision making. We assess the ability of early continuous EEG monitoring at the intensive care unit (ICU) in patients with traumatic brain injury (TBI) to predict long term clinical outcome and evaluate its complementary value to current clinical standards. We performed continuous EEG measurements in patients with moderate to severe TBI during the first week of ICU admission. We assessed the Extended Glasgow Outcome Scale (GOSE) at 12 months, dichotomized into poor (GOSE 1–3) and good (GOSE 4–8) outcome. We extracted EEG spectral features, brain symmetry index, coherence, aperiodic exponent of the power spectrum, long range temporal correlations, and broken detailed balance. A random forest classifier using feature selection was trained to predict poor clinical outcome based on EEG features at 12, 24, 48, 72 and 96 h after trauma. We compared our predictor with the IMPACT score, the best available predictor, based on clinical, radiological and laboratory findings. In addition we created a combined model using EEG as well as the clinical, radiological and laboratory findings. We included hundred-seven patients. The best prediction model using EEG parameters was found at 72 h after trauma with an AUC of 0.82 (0.69–0.92), specificity of 0.83 (0.67–0.99) and sensitivity of 0.74 (0.63–0.93). The IMPACT score predicted poor outcome with an AUC of 0.81 (0.62–0.93), sensitivity of 0.86 (0.74–0.96) and specificity of 0.70 (0.43–0.83). A model using EEG and clinical, radiological and laboratory parameters resulted in a better prediction of poor outcome (p < 0.001) with an AUC of 0.89 (0.72–0.99), sensitivity of 0.83 (0.62–0.93) and specificity of 0.85 (0.75–1.00). EEG features have potential use for predicting clinical outcome and decision making in patients with moderate to severe TBI and provide complementary information to current clinical standards.

## Highlights

• Prediction of clinical outcome for severe traumatic brain injury remains challenging.
• We investigated the prediction accuracy of early EEG for traumatic brain injury.
• Early EEG provides complementary predictive information to current clinical standards.

## 1 Introduction

Clinical decision making at the intensive care unit (ICU) for patients with moderate to severe traumatic brain injury (TBI) remains challenging (Carney et al., 2017, Lingsma et al., 2010). A composed score based on clinical, radiological and laboratory findings (International Mission for Prognosis And Clinical Trial Design (IMPACT) predictor) is currently the best available predictor for clinical outcome in patients with TBI (Murray et al., 2007). However, the IMPACT predictor only takes into account information from admission and is not sensitive to effects resulting from secondary brain injury. There is an ongoing need for ideally non-invasive prognostic markers that could boost the performance of clinical prediction models to aid in clinical decision making and to inform relatives of patients about expectations (Maas et al., 2017).

Continuous electroencephalography (EEG) monitoring shortly after admission has been proposed as add-on marker in patients with moderate to severe TBI (Turgeon et al., 2017), not only to rule out potential nonconvulsive status epilepticus (Ronne-Engstrom and Winkler, 2006, Vespa, 2005), but also to predict long term clinical outcome (Vespa et al., 2002, Hebb et al., 2007, Tolonen et al., 2018, Mikola, et al., 2015, Kane et al., 1998, Thatcher et al., 2001). Continuous EEG monitoring could potentially track effects of secondary brain injury. Poor clinical outcome has been linked to slowing of the EEG with decrease in alpha power (Tolonen et al., 2018, Kane et al., 1998, Haveman et al., 2019) and lower variability in alpha power (Vespa et al., 2002, Hebb et al., 2007, Haveman et al., 2019). However, small to moderate sample size or inclusion of single EEG features capturing only a fraction of information of ongoing EEG activity (Lee et al., 2019) has hindered to underscore the potential relevance of continuous EEG monitoring in clinical practice.

Changes of EEG spectral power in TBI have been well described (Thatcher et al., 1989), though little is known about the role of time-resolved characteristics of EEG oscillations in TBI. Two ways to describe the temporal evolution of EEG oscillations are the estimation of long range temporal correlations (LRTC) and’broken detailed balance’ (Linkenkaer-Hansen et al., 2001, Ton and Daffertshofer, 2016, Battle et al., 2016, Perl et al., 2021). These methods allow to estimate the amount of memory within EEG oscillations and the amount of entropy production in the brain respectively. These abstract concepts have been applied to neurophysiological data (Thiery et al., 2018, Perl et al., 2021), though little is known whether these abstract concepts are able to predict clinical outcome. Another novel way to obtain comprehensive information of EEG activity is the quantification of the aperiodic part of the power spectrum (He, 2014). The shape of this part of the power spectrum is believed to relate to inhibition excitation ratios (Donoghue et al., 2020), for which there are hints of impairment in TBI (Ding et al., 2011).

In the present study, we evaluate the use of continuous and early EEG monitoring to predict long term clinical outcome in patients with TBI as an add-on to the current clinical standards using state-of-the art EEG measures and machine learning algorithms for outcome classification. We build on previous work that reported interim results (Haveman et al., 2019). We now report the full dataset, we add several new EEG features (e.g. aperiodic part of the power spectrum, LRTC, broken detailed balance) and perform cross-validation.

## 2 Methods

### 2.1 Study population

All patients with moderate to severe TBI were included between 2013 and 2021 in two prospective cohorts at Medisch Spectrum Twente, a large teaching hospital and trauma center. Inclusion criteria were patients with moderate to severe TBI (Glasgow Coma Scale ≤ 12 at the trauma location or emergency department) admitted to the ICU with an expected stay of >24 h. Exclusion criteria were trauma following or combined with severe circulatory failure (cardiac arrest), earlier TBI or stroke without full recovery, progressive brain illness (tumor, neurodegenerative disease), limited life expectancy (<6 months) prior to TBI. The institutional review board waived the need for written informed consent as EEG is part of our routine care. Verbal consent was asked for outcome assessment in the first cohort from 2013 to 2016. In the second cohort from 2017, patients also underwent other measurements (including TCD and/or CT perfusion). Since this study was more extensive in this cohort, written informed consent was obtained from the legal representative and (if possible) from the patient during follow-up.

### 2.2 Outcome assessment and IMPACT predictors

The extended Glasgow Outcome Scale (GOSE) was used as primary outcome score, which was obtained after phone inquiry at 12 months after admission. GOSE scores were dichotomized into poor outcome (GOSE 1–3) and good outcome (GOSE 4–8). For each patient, all information to estimate the IMPACT score was collected, i.e. Glasgow coma score at admission, pupil response, hypotension (mean arterial pressure *<* 90 mmHg), hypoxia (arterial oxygen saturation *<* 90 %), CT-brain findings according to the Marshall classification (Marshall et al., 1992), glucose and hemoglobin levels at admission. Additional clinical information was also obtained, which included administration of sedative (e.g. midazolam, propofol, barbiturates) and anti-epileptic drugs, his- tory of seizures, neurosurgical interventions (intracranial pressure monitoring, craniotomy), and total length of ICU admission.

### 2.3 EEG preprocessing

Continuous EEG recordings were commenced once patients were admitted to the ICU and continued until seven days after injury. Nineteen electrodes (either silver/silver chloride cup or subdermal wire) were placed according to the 10–20 International System. A limited set of electrodes was placed in six subjects (Cz, O1, O2, C3, C4, T3, T4, Fp1, Fp2). A Neurocenter EEG system with Refa or SAGA amplifiers (TMSi, Netherlands) was used, recording at a sample frequency of 256 Hz. At 12, 24, 48, 72 and 92 h after TBI 30 min of EEG data was further preprocessed using a zero-phase sixth-order Butterworth bandpass filter of 0.1–40 Hz. We used a semi-automated algo- rithm to detect and remove artifacts within windows of 10 s in the common average (Ruijter et al., 2018, Tjepkema-Cloostermans et al., 2017). Artifacts included empty channels, channels with large peaks or noise (amplitude ≥ 200 µ*V* or ≤−200 µ*V* and variance ≥1400 µ*V* ^2^ or ≤1 µ*V* ^2^), or muscle artifacts. In addition, we used independent component analysis to detect and remove the ECG artefact after visual inspection of individual components (Hyvarinen 1999). After pre-processing, 30 min around time of interest was divided into three segments of 10 min. EEG features were computed for three 10 min segments to provide our machine learning algorithm with more data.

### 2.4 EEG features

The selection of EEG features was based on previous work. We quantified a similar set of EEG features as in our previous work (Haveman et al., 2019). In addition, we quantified the aperiodic component (1/f part) of the power spectral density (Donoghue et al., 2020), LRTC (Ton and Daffertshofer 2016) and the extent of broken detailed balance (Lynn, et al., 2021, Perl et al., 2021). We calculated sixteen features in total. Seven out sixteen features were calculated for every frequency band separately; delta (0.5–4 Hz), theta (4–8 Hz), alpha (8–13 Hz), beta (10–20 Hz). EEG features were only calculated if the recording at time of interest (e.g. 12, 24, 48, 72, 96 h after trauma) included non-flat channels (defined as amplitude variance ≤ 0.1 µ*V* ^2^). EEG preprocessing and feature calculation were performed using Matlab R2020a (The MathWorks, Inc., Natick, MA).

*Power spectral features:* We estimated the power spectral density (PSD) for every segment of 10 min of data using the Welch’s method with windows of 10 s with no overlap. The *total power* was defined as the sum across all frequency bins. *Absolute power* was calculated by integration of the PSD within each frequency band. *Relative power* was further defined as the ratio between absolute power in each frequency band and total power. The *alpha/delta ratio (ADR)* was quantified as the ratio between absolute alpha and delta power, more specifically (alpha - delta)/(alpha + delta). The *spectral edge frequency 90 %* was defined as the frequency for which 90 % of the total power lies below this cut-off. *Variability* was computed for every frequency band separately and defined in terms of the ratio of the median absolute deviation to the median power in each frequency band (Tolonen et al., 2018, Haveman et al., 2019).

*Brain symmetry index:* The pairwise derived brain symmetry index (BSI) was used to estimate the symmetry of power between pairs of electrodes from left and right hemispheres (Sheorajpanday et al., 2009), expressed as a value between 0 (symmetric) and 1 (highly asymmetric). BSI was calculated for the frequency range 0.5–20 Hz.

*Coherence:* Synchronization between channels was quantified by calculation of coherence (Nunez et al., 1997). This was defined as the mean of all magnitude squared coherences between all possible combinations of channels using a Hann window of 4 s and an overlap of 2 s, resulting in a value between 0 (no synchronization) and 1 (full synchronization).

*Shannon entropy:* The entropy was defined in terms of the Shannon entropy (Shannon 1948), where higher entropy corresponds to a less predictable system.

*Regularity:* Regularity is a measure for the continuity of the EEG based on the variance of the amplitude of the signal. Regularity is normalized between 0 and 1, where a higher value indi- cates a signal with more regular amplitude (Tjepkema-Cloostermans et al., 2013).

*Aperiodic component:* the shape of the PSD can be characterised by an aperiodic compo- nent (1*/f* ^α^ part) superimposed by peaks corresponding to periodic components (He 2014). The *off-set* and *exponent α* of the (1*/f* ^α^ part) part were estimated using the approach described in (Donoghue et al., 2020) and using their corresponding’FOOOF’ toolbox in Python in conjunction with the Matlab wrapper (https://github.com/fooof-tools/fooof).

*Long range temporal correlations:* The existence of long range temporal correlation was as- sessed using detrended fluctuation analysis (DFA) using the implementation of (Daffertshofer et al., 2018, Ton and Daffertshofer, 2016), (Matlab code: https://github.com/marlow17/FluctuationAnalysis). DFA analysis was performed for each individual frequency band. The analytical signal of band- pass filtered data in each frequency band was extracted using the Hilbert transform, from which both the phase and amplitude envelope dynamics were estimated. Either the sum of the amplitude envelopes or the global phase dynamics (i.e. Kuramoto order parameter) were used as input to DFA analysis. DFA quantifies the autocorrelation of a cumulative signal and results in estimation of the Hurst exponent, where H = 0.5 corresponds to an uncorrelated process with no memory and H *>* 0.5 to the presence of long range temporal correlations and memory. We used windows ranging from the duration of one oscillation (depending on the frequency band) to one fourth of the recording time to compute the autocorrelation function.

*Broken detailed balance:* The extent of nonequilibrium brain dynamics was characterised using the approach as outlined in (Lynn et al., 2021) (Matlab code: https://github.com/ChrisWLynn/Broken detailed balance). Detailed balance refers to the concept that transitions between two states are pairwise balanced, i.e. there is no preferred transition from state A to B or vice versa. Broken detailed balance refers to the violation of this concept. Brain states can be estimated from the first two principal components of the band-pass filtered EEG data (separate analysis is performed for every frequency band). A transition probability matrix can be estimated based on the current and future magnitudes of the first two principal components. The asymmetry of this transition probability matrix or the *entropy production* and the curl of the corresponding probability flux (Perl et al., 2021) can be defined as means to describe broken detailed balance.

### 2.5 Machine learning classification

Prediction models were first trained using merely EEG features, secondly using both EEG features and IMPACT parameters. The latter was also compared to the IMPACT score based on the logistic regression model of the original work (Steyerberg et al., 2008). A random forest classifier was used to solve a binary classification problem and used to predict poor clinical outcome (i.e. GOSE 1–3) (Breiman, 2001). The random forest classifier is a supervised method that uses multiple (bootstrap- aggregated or bagged) decision trees to solve a classification problem. Every tree is constructed using a subset of features and samples. Classification results from the majority of votes from the different decision trees, which allows for minimization of overfitting. Out-of-bag error was used to find the optimal number of trees, which was set to 200. The predictive value was evaluated using 5-fold cross-validation, in which 80 % of the data were used as training set and 20 % of the data as test set. Classification results were evaluated using the area under the curve (AUC) of the receiver operator characteristic (ROC) curve, and using specificity and sensitivity. Results for only the test set are reported. Data from different time points (12, 24, 48, 72 and 96 h after trauma) were separately fed to the random forest classifier using all EEG features. A specific form of data augmentation was employed. For every time point, EEG features for every 10 min of data (3 × 10 min) were used as input data points. Feature selection was subsequently performed for *t* with the highest AUC using a backward elimination approach. A prediction model was trained using all features and for every subsequent step, the feature with the lowest importance from the previous step was eliminated and the prediction model retrained. This allowed us to keep track of the AUC.

as a function of numbers of features eliminated. A substantial drop in the AUC (based on visual inspection) was used as stop criterion and allowed us to determine the eventual number of features. After feature selection, data from two consecutive time points *t* were aggregated and fed into the classifier, which was also repeated for data from three consecutive time points. Differences between model predictions were assessed using the McNemar’s test (*p <* 0*.*05 was assumed to correspond to statistical significance). Correction for multiple tests was performed by the false discovery rate (Benjamini and Hochberg, 1995).

## 3 Results

A total of hundred-seven patients with moderate to severe traumatic brain injury were included. Three patients were excluded due to lack of GOSE outcome scores (n = 1), concurrent cardiac arrest (n = 1) and lack of EEG data for *t* ≤ 96 h (n = 1), resulting in hundred-four patients for further analysis. Table 1 shows the baseline characteristics.

**Table 1:** Patient characteristics.

Descriptive	Poor outcome GOSE 1–3 (n = 55)	Good outcome GOSE 4–8 (n = 49)	p-value
Sex/females (%)	15 (27 %)	14 (29 %)	…
Age in years (median (IQR))	59 (46–70)	43 (13–57)	*<*0.001^2^
History of seizures (n)	2	1	
Alcohol usage (n)	3	0	
ICU stay in days since trauma (median (IQR))	10 (5–15)	13 (6–22)	…
EEG start in hours after trauma (median (IQR))	12.3 (4.5–22.7)	12.2 (6–19.3)	…
EEG recording time in hours (median (IQR))	119.6 (64.6–160.9)	93.2 (40.5–146)	…
Intracranial Pressure Monitoring (n, %)	29 (53 %)	17 (35 %)	0.02^1^
Decompressive craniotomy (n, %)	13 (24 %)	9 (18 %)	…
Administration of sedative drugs (n, %)	55 (100 %)	49 (100 %)	…
Midazolam (n, %)	44 (80 %)	28 (57 %)	…
Propofol (n, %)	53(96 %)	48 (98 %)	…
Thiopental (n, %)	3 (5 %)	3 (6 %)	…

1 Statistical result of the Chi square test. ^2^Statistical result of the Mann-Whitney U test….… = *p >* 0*.*05.

### 3.1 Model and feature selection

**Fig. 1:**
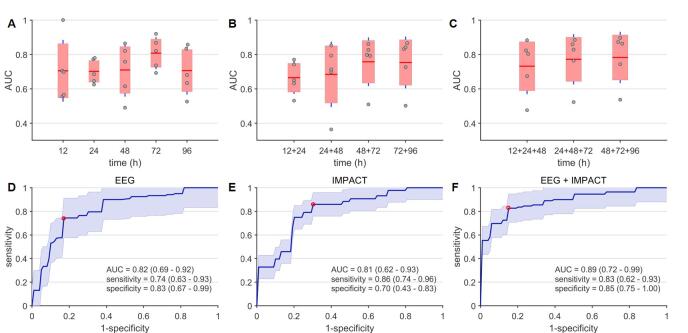
Prediction accuracy of poor clinical outcome for different models applied to the test set. Panel A-C show prediction accuracy in terms of AUC values for models based on EEG data from one, two or three consecutive time points with fair to good prediction accuracy. Panel D-F show the ROC curves for the model based on EEG features alone (D), the IMPACT score (E) and the model based on a combination of EEG features and IMPACT parameters (F). There is significant improvement in prediction accuracy when EEG features and IMPACT parameters are combined.

As first step, we predicted clinical outcome based on age and all EEG features from one time point (e.g. 12, 24, 72 or 96 h after trauma). Figure S1 shows a boxplot diagram for the AUC values for every time point and different folds of the 5-fold cross-validations. For most pair of time points there was no significant difference in prediction accuracy. However, the model for 72 h after trauma resulted in significantly better predictions than the models for 12 or 24 h after trauma (McNemar’s’ test *p <* 0*.*001). Feature selection for this time point yielded 16 EEG features (see Figures S2 and S3), which were used for all further analysis. Feature selection for this time point was not critical since re-doing feature selection for EEG data from 48 h after trauma resulted in the same selected features. Note that most contributing features to predict poor clinical outcome were classical power spectral measures, e.g. absolute and relative power in different frequency bands, along with total power, the BSI, coherence, the aperiodic component of power spectra, variability in the alpha band and the Hurst exponent in the delta band. The best EEG based model after feature selection was found for 72 h after trauma with a mean AUC of 0.82 (range 0.69–0.92) (see Fig. 1A), a specificity of 0.83 (range 0.67–0.99) and a sensitivity of 0.74 (range 0.63–0.93) (Fig. 1D). Again, the model at 72 h after trauma showed significant better predictions than the models for 12 or 24 h after trauma (McNemar’s test *p <* 0*.*001). Combining EEG features from consecutive time points (e.g. 12 + 24 h) did not result in higher AUC compared to single time points (see Fig. 1B; McNemar’s test *p >* 0*.*05). Though model performances for 48 plus 72 h after trauma and 72 plus 96 h after trauma was better compared to model performance for 12 plus 24 h after trauma (McNemar’s test *p <* 0*.*001). Pooling EEG features from three consecutive time points did not improve performance (McNemar’s test *p >* 0*.*05, see Fig. 1C).

### 3.2 Combination of EEG, clinical and radiological parameters

**Fig. 2:**
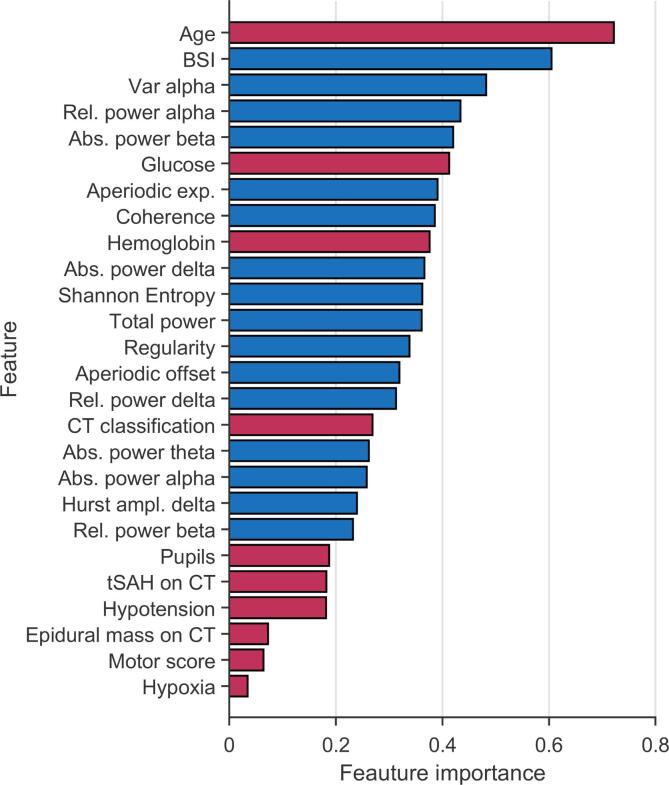
Feature importance for best model based on a combination of IMPACT parameters and selected set of EEG features. Red bars correspond to IMPACT parameters and blue bars to EEG features. The five most relevant features are age, BSI, variability in the alpha band, relative power in the alpha band and absolute power in the beta band. Abbreviations: brain symmetry index (BSI), absolute (abs.), relative (rel.), exponent (exp.), amplitude (ampl.), computer tomography (CT), traumatic subarachnoidal hemorrhage (tSAH).

We evaluated the IMPACT score in the same validation sets as the EEG model. The IMPACT predictor predicted outcome with a mean AUC of 0.81 (range 0.62–0.93), with a sensitivity of 0.86 (range 0.74–0.96) and a specificity of 0.70 (range 0.43–0.83, see Fig. 1E). Model performance significantly improved by combining EEG (EEG features from 72 h after trauma) and the clinical, radiological and laboratory parameters from the IMPACT score (Fig. 1F) (McNemar’s test *p <* 0*.*001). This best performing model predicted poor outcome with an AUC of 0.89 (range 0.72–0.99), with a sensitivity of 0.83 (range 0.62–0.93) and a specificity of 0.85 (range 0.75–1.00). Fig. 2 shows the feature importance for this combined model. Classical EEG spectral measures, the BSI and age contributed most strongly to the ability to predict poor outcome. Out of IMPACT predictors age, glucose and hemoglobin at admission had strong discriminative power, while CT findings, motor score, pupils, hypoxia, hypotension had only moderate relevance.

## 4 Discussion

We have demonstrated using a machine learning approach that early EEG measurements strongly predict poor outcome in patients with moderate to severe traumatic brain injury. EEG parameters had the same or maybe even slightly better predictive power compared to current clinical standards (IMPACT score). A combined model with both EEG and IMPACT predictors resulted in a prediction with both high sensitivity 0.83 and specificity 0.85 and outperformed a model based on the IMPACT score alone.

So far, the role of EEG monitoring in patients with moderate to severe traumatic brain injury has been limited to the assessment of nonconvulsive status epilepticus or the background rhythm or to guide barbiturate therapy. Previous EEG studies in this population have been unable to provide sufficient support to extend this role to prediction of long term clinical outcome (Tolonen et al., 2018, Jasper et al., 1940, Williams, 1941, Claassen et al., 2019, Moulton et al., 1988, Kane et al., 1998, Vespa et al., 2002, Hebb et al., 2007), due to various reasons. While most EEG studies have been carried out in the subacute to chronic stage (Nuwer et al., 2005), fewer studies have attempted to evaluate the use of EEG monitoring in the acute stage at the ICU (Lee et al., 2019, Tolonen et al., 2018, Claassen et al., 2019, Moulton et al., 1988, Kane et al., 1998, Vespa et al., 2002, Hebb et al., 2007). However, in contrast to our work, most of this previous work has relied on only a few and merely classical EEG parameters (Hebb et al., 2007, Tolonen et al., 2018, Lee et al., 2019), classical statistical tests, mere visual inspection ((Jasper et al., 1940, Williams, 1941) or were constrained by limited sample size (Tolonen et al., 2018, Mikola, et al., 2015, Moulton et al., 1988). The combination of a fairly large sample size, multiple EEG features (including more advanced metrics) and a machine learning approach resulted in the current findings that support a more dominant role of EEG monitoring in the prediction of clinical outcome in these patients. Our findings are in agreement with our previous study in a subgroup of our TBI population (Haveman et al., 2019). Though in our previous work prediction accuracy was lower probably due to a smaller sample size and the inclusion of additional EEG features in the current work.

Our current work hints to more importance of EEG features compared to IMPACT parameters alone. The combined EEG and IMPACT model revealed that apart from age, most contributing features to predict poor outcome were EEG features. An important reason for the good prediction accuracy of EEG features is the potential sensitivity to secondary injury (Lewine et al., 2019). Secondary injury is believed to be at least equally important in explaining neurological outcome compared to primary injury (Sharp et al., 2014, Toth, et al., 2016). IMPACT parameters on the other hand are a reflection of primary injury and hence a combination of measures sensitive to primary (IMPACT) and secondary (EEG) injury may lead to better prediction of outcome. Another reason for the good prediction accuracy of EEG is its sensitivity to track synaptic damage. Recent preclinical and animal work suggest that secondary damage is significantly reflected in synaptic damage (Jamjoom et al., 2021).

We have analyzed the timing of the EEG measurement after trauma. The best performing model was found for EEG data obtained around 72 h after trauma, though no difference in performance was found compared to data from 48 or 96 h after trauma. EEG data obtained around 72 h performed better in predicting clinical outcome compared to data from 12 and 24 h after trauma. This suggest that continuous EEG recording seem to be most opportunistic at least 24 h after trauma or later. For clinical practice this would indicate that EEG measurement for consecutive days is not necessary.

The best EEG prediction model showed strong discriminative ability for the brain symmetry index (BSI), total power, absolute power in the delta band, variability of power in the alpha band, the offset of the aperiodic component of the power spectrum. Moderate to strong relevance were found for the remaining classical power spectral features, but also for coherence, regularity, the exponent of the aperiodic component of the power spectrum and the Hurst exponent in the delta band. Characteristics of the power spectrum seem to be the most important to predict outcome in TBI, as the bulk of these measures are descriptors of or related to the power spectrum. A few mea- sures warrant some further discussion. The most important feature, the BSI, indicates that strong asymmetry contributes strongly to prediction of poor clinical outcome. A non-classical measure, the aperiodic component of the power spectrum provides complementary predictive ability to the model, and hence captures information not merely explained by band specific power or alpha/delta ratio. Out of the more advanced measures, long range temporal correlations and broken detailed balance, only the presence of long range temporal correlations or memory in the delta band contributed to the prediction of poor outcome. Despite the fact that measures of broken detailed balance have been able to discriminate the awake state and loss of consciousness (Perl et al., 2021), our current work shows that these measures do not provide complementary information to predict clinical outcome in TBI.

The prediction accuracy of the IMPACT score in the current dataset was in line with previous work, a recent systematic review on IMPACT based prediction of poor outcome in TBI reported a mean AUC of 0.79 (Dijkland et al., 2020), which was around the same value as in our work (AUC 0.81). The most relevant IMPACT features apart from age were hemoglobin and glucose at admission, and to a lesser extent CT classification, motor score and pupillary response. Other IMPACT parameters such as traumatic subarachnoidal hemorrhage, epidural mass on CT were less relevant for our models. Feature importance of these results are not entirely in line with the original work on the IMPACT prediction (Steyerberg et al., 2008, Lingsma et al., 2010), where motor score and pupillary response were found to contain the most prognostic information, though these were of moderate importance in our dataset. However our current feature importance cannot be compared vis-a-vis with the initial work on the IMPACT prediction given two very different sets of features (EEG versus no EEG) and the use of classical statistics versus machine learning. Our work is in agreement with previous work (Haveman et al., 2019, Lee et al., 2019), both emphasizing increased predictive performance when IMPACT parameters and EEG features are combined. In contrast to our earlier work reporting interim results (Haveman et al., 2019), we have now showed that a model combining IMPACT and EEG features outperforms the IMPACT score alone.

## 5 Conclusion

In sum, in a large prospective cohort of patients with moderate to severe traumatic brain injury, we showed that early continuous EEG monitoring at the ICU predicts long term poor clinical outcome with fair to good sensitivity and specificity. The addition of EEG features to current standards including clinical, radiological and laboratory parameters significantly improved prediction of poor clinical outcome. Hence, current findings suggest to extend prediction parameters in moderate to severe traumatic brain injury with quantitative EEG assessment. A future study using early EEG monitoring in an independent study population of patients with moderate to severe traumatic brain injury could provide further support.

## Declaration of Competing Interest

The authors declare that they have no known competing financial interests or personal relationships that could have appeared to influence the work reported in this paper.

## Data Availability

Data will be made available on request.
