# Supplementary material for: Early EEG monitoring predicts clinical outcome in patients with moderate to severe traumatic brain injury
Source: Neuroimage Clin. 2023 Feb 14;37:103350. doi: 10.1016/j.nicl.2023.103350 (PMC9984683; doi:10.1016/j.nicl.2023.103350)
Supplement: Supplementary data 1 [file mmc1.pdf]

Supplementary material: Early EEG monitoring predicts clinical outcome in patients with moderate to severe traumatic brain injury

Prejaas Tewarie[1,2], Tim M.J. Beernink[2], Carin J. Eertman-Meyer[2], Alexander D. Cornet[3], Albertus Beishuizen[3], Michel J.A.M. van Putten[1,2], and Marleen C. Tjepkema-Cloostermans[1,2]

[1]Clinical Neurophysiology group, University of Twente, Enschede, The Netherlands
[2]Department of Neurology and Clinical Neurophysiology, Medisch Spectrum Twente, Enschede, The Netherlands
[3]Intensive Care Center, Medisch Spectrum Twente, Enschede, The Netherlands

October 2, 2022

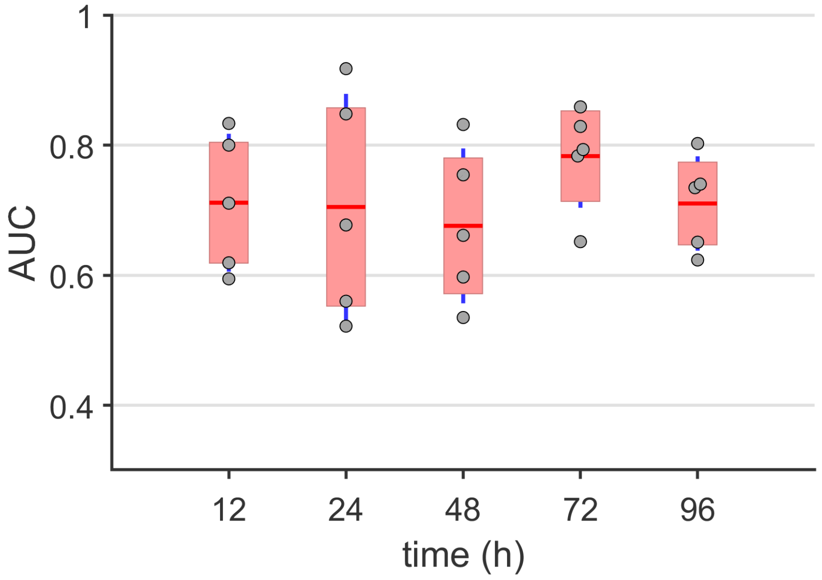

Figure 1: Prediction accuracy of poor clinical outcome for models trained for different times. Only EEG features were used for the training and all EEG features (prior to feature selection) were used.

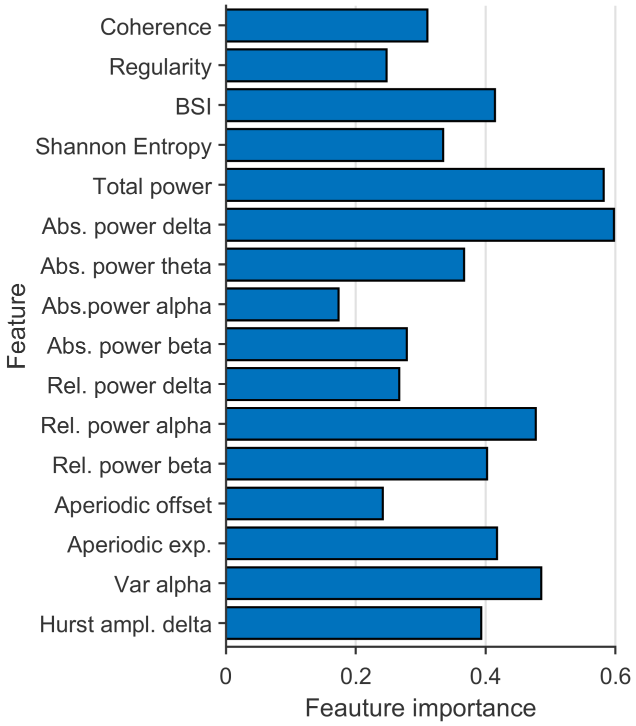

Figure 2: Feature importance for best model based on only EEG features. This EEG based model was trained for $t = 72$ h. The most important features were power spectral measures, e.g. absolute and relative power in different frequency bands, along with total power, the BSI, coherence, the aperiodic component of power spectra, variability in the alpha band and the Hurst exponent in the delta band

.

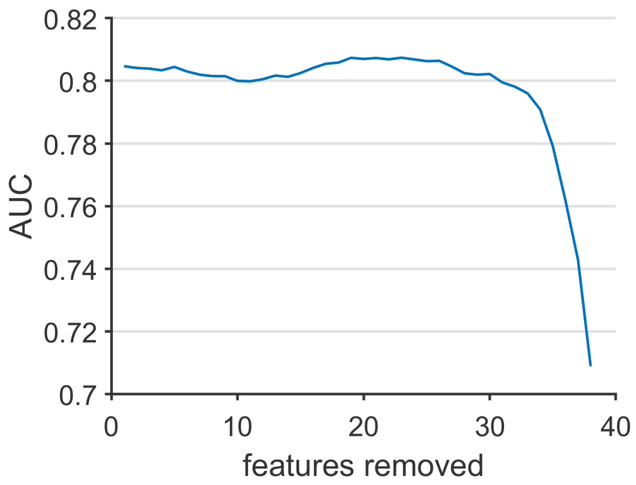

Figure 3: Feature selection was performed for t with the highest AUC ($t = 72$ h) using a backward elimination approach. A prediction model was trained using all features and for every subsequent step, the feature with the lowest importance from the previous step was eliminated and the prediction model retrained. The AUC as a function of number of removed features is illustrated.

.
